# A Unique Interplay of Multiple Predisposing Factors Culminating in a Catastrophic QT Prolongation

**DOI:** 10.7759/cureus.7757

**Published:** 2020-04-21

**Authors:** Vrinda Vyas, Alisha Khan, Gowthami Kanagalingam, Luna Bhatta

**Affiliations:** 1 Internal Medicine, State University of New York (SUNY) Upstate Medical University, Syracuse, USA; 2 Medicine, State University of New York (SUNY) Upstate Medical University, Syracuse, USA; 3 Cardiology, Electrophysiology, State University of New York (SUNY) Upstate Medical University, Syracuse, USA

**Keywords:** torsades de pointes, sudden cardiac death, icd, long qt syndrome

## Abstract

Implantable cardioverter-defibrillators (ICDs) are used in patients without a reversible cause for long QT syndrome (LQTS) and secondary prevention in patients with LQTS-associated sudden cardiac arrest. We present a female patient with multiple reversible factors for QT prolongation, including the use of antidepressants, antidiarrheals, antiemetics, and antihistamines; chronic malabsorption from bariatric surgery; probable Gitelman syndrome and urinary losses of electrolytes, causing QT prolongation which leads to polymorphic ventricular tachycardia and a successfully resuscitated cardiac arrest. Our patient also had history suggestive of probable congenital LQTS with multiple childhood syncopal episodes and a history of seizures in first-degree relatives, further justifying the placement of an ICD. Also, this case gives us an opportunity to delve into the risks of catastrophic QT prolongation in the morbidly obese population undergoing bariatric surgery.

## Introduction

Long QT syndrome (LQTS) is a rare cardiac channelopathy characterized by a prolonged QT interval on the electrocardiogram (EKG). It is associated with syncope and sudden death due to torsades de pointes and ventricular fibrillation. LQTS may be either congenital or acquired. Acquired LQTS usually results from drug therapy or electrolyte imbalances such as hypokalemia, hypomagnesemia, and bradycardia. The primary symptoms in patients with LQTS include palpitations, syncope, seizures, and sudden cardiac death. The management of patients with LQTS consists of life-style modification, beta-blockers, left cardiac sympathetic denervation (LCSD), and cardioverter-defibrillator (ICD) implantation.

## Case presentation

**Figure 1:**
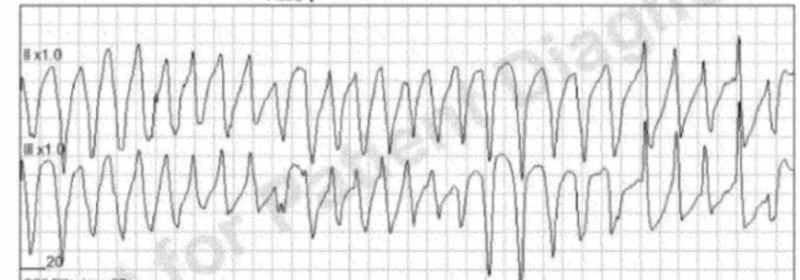
Telemetry strip obtained by EMS demonstrating torsades EMS=emergency medical services

We present a case of a 57-year-old female who called emergency medical services (EMS) for episodic dizziness of one-day duration. When paramedics arrived, the patient was alert and oriented but appeared to be in discomfort; her vitals were within limits. While the patient was being transported in the ambulance, she started experiencing dizziness and was noted to have torsades de pointes on the telemetry strip. The patient remained alert and oriented, with a palpable pulse during the multiple runs of ventricular tachycardia. She was given normal saline bolus and 150 mg of amiodarone in the ambulance. On arrival at the emergency department (ED), the patient was noted to have intermittent non-sustained polymorphic VT in the setting of a prolonged QTc interval of 520 msec (Figure 1). Potassium and magnesium resulted low at 2.2 mmol/L and 1.1 mg/dL, respectively. Her electrolytes were vigorously repleted and an amiodarone drip was started, following which the patient had a ventricular fibrillation arrest and was cardioverted to sinus rhythm temporarily. She continued to have polymorphic VT and was started on lidocaine drip. The patient was then admitted to the cardiac intensive care unit (CCU).

The patient had a significant history of multiple syncopal episodes since 2015, which were initially attributed to orthostatic hypotension and the patient was started on fludrocortisone, but when she continued to have syncopal episodes, a loop recorder was placed in June 2017 by her cardiologist. On an extensive review of history, it was established that the patient had been having severe diarrhea and nausea among other gastrointestinal symptoms since undergoing gastric bypass surgery in 2013. The patient reported using Zofran and Imodium as needed up to even four to six times a day. She also reported a history of depression for which she was on escitalopram. She also reported the use of hydroxyzine for allergies and omeprazole for gastroesophageal reflux disease (GERD). The patient recalled a history of multiple episodes of syncope as a child, which was never evaluated and had subsided on its own in her teenage years. The patient denied any family history of sudden cardiac deaths or syncope but noted the history of seizures in her sister. She was noted to have sinus bradycardia with prolonged QT in her last outpatient visit with her primary cardiologist but electrocardiograms (EKGs) before that had been completely unremarkable, with normal QTc. Her primary cardiologist confirmed her loop recorder showed nonsustained episodes of torsades des pointes while the patient was at home and experiencing dizzy spells before calling EMS.

**Figure 2:**
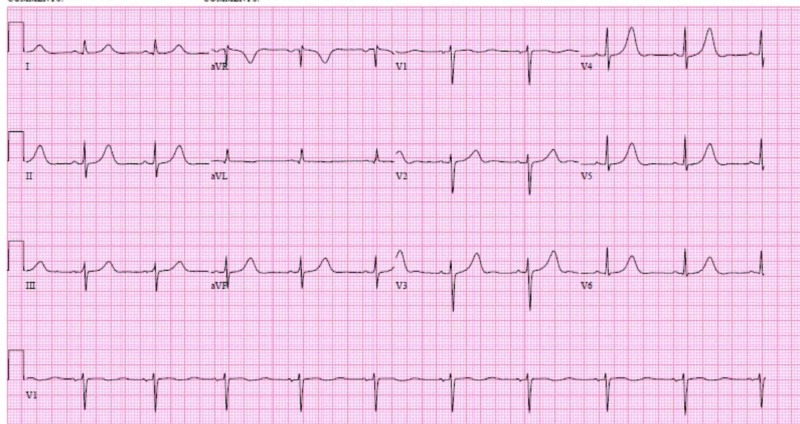
EKG after correction of patient's reversible QTc prolonging factors, prior to ICD placement EKG=electrocardiogram; ICD=implantable cardioverter defibrillator

In the CCU, the patient’s QT-prolonging medications were stopped. Her electrolytes were vigorously monitored and replaced. Labs for genetic testing for congenital long QT syndrome were sent. Lidocaine drip was stopped after two days when the patient’s EKG became largely unremarkable except for the presence of sinus bradycardia. See Figure 2.

**Figure 3:**
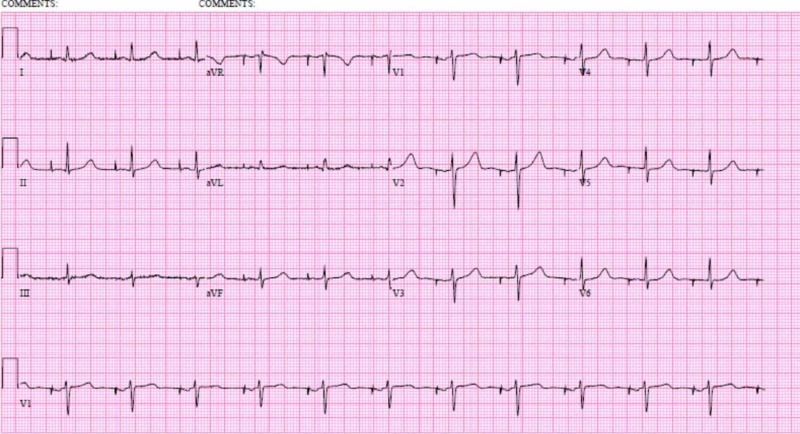
Patient's EKG after ICD placement, on the day of discharge EKG=electrocardiogram; ICD=implantable cardioverter defibrillator

The patient continued to have hypomagnesemia and hypophosphatemia despite adequate replacement. Her urine studies showed abnormal urinary magnesium, phosphate, and chloride losses and were suggestive of either surreptitious diuretic use or Gitelman's syndrome. Hence, nephrology was consulted. The elevated urine chloride was more consistent with Gitelman’s syndrome, but the patient’s diarrhea was also complicating the overall electrolyte homeostasis. Hence, the patient was started on spironolactone 12.5 mg daily as per nephrology recommendations and will be followed up by nephrology as an outpatient where they plan on repeating the urine studies. Our patient’s long QT was attributed to the many QT-prolonging drugs she was taking, including escitalopram, loperamide, ondansetron, and hydroxyzine, her history of bariatric surgery leading to chronic malabsorption and electrolyte imbalance, chronic omeprazole use further causing hypomagnesemia, suspected Gitelman’s syndrome, and a very high suspicion for underlying congenital forms of long QT syndrome (from the childhood history of syncope and the history of possible seizures or syncope in her sister). Hence, she underwent implantable cardioverter-defibrillator placement and was discharged home the next day in a stable condition (Figure 3). In a month, the patient’s genetic testing resulted and ruled out congenital long QT syndrome, thereby confirming a diagnosis of acquired long QT.

## Discussion

LQTS is caused by a disturbance in myocardial repolarization, which is characterized by a prolonged QT interval on EKG that leads to ventricular arrhythmias and sudden cardiac death (SCD) [1]. The main symptoms in patients with LQTS include syncope, seizures, cardiac arrest, and SCD [1]. LQTS may be congenital or acquired, or a combination of both, as some patients who develop acquired LQTS may have an inherited predisposition for a prolonged QT [1]. Multiple genes that help in the production of transmembrane ion channels have been implicated in congenital LQTS; KCNQ1 (LQT1), KCNH2 (LQT2), and SCN5A (LQT3) are the most common ones [1-2]. For all patients in whom congenital LQTS is suspected, the Schwartz score is calculated to better estimate the clinical likelihood of a congenital LQTS. The Schwartz score takes into consideration the EKG findings (including QTc length, presence of torsades, bradycardia), clinical history (including the history of recurrent syncope, congenital deafness), and family history (of SCD, definite LQTS) to estimate the probability of LQTS in a given patient. The case we present had a Schwartz score of 4. When a patient satisfies a high probability Schwartz score (≥3.5 points), the likelihood of a positive LQTS genetic test is approximately 80% [3].

Our patient had multiple factors causing a long QT interval including the use of multiple drugs (such as escitalopram, loperamide, ondansetron, hydroxyzine ), gastric bypass leading to malabsorption, chronic diarrhea leading to persistent hypokalemia and hypomagnesemia, possible renal losses of electrolytes (Gitelman’s syndrome was suspected) with chronic sinus bradycardia causing further QTc prolongation. Before our patient's genetic testing resulted, it was thought that our patient might have either congenital LQT1 or she might have had a concealed form of congenital LQTS in which a mutation in one of the LQTS genes is clinically inapparent until the patient is exposed to other predisposing factors. Our patient's Schwartz score was 4 as mentioned above, further increasing the likelihood of congenital LQTS rather than an acquired one. To our surprise, the patient did not have any mutation in the 42 genes that were studied in the genetic screening test she underwent. Although, it is important to note that about 20% of those who meet the clinical criteria for LQTS do not have any mutations in the commonly studied genes and fall in the genotype negative category.

To the multitude of predisposing factors behind our patient’s prolonged QT, the clinical question our team was faced with while treating this patient was whether an ICD should be placed or not. Studies have proved that the overdiagnosis and overtreatment of LQTS with unnecessary ICD placements are associated with multiple complications such as inappropriate shocks, infection, device malfunction among others [4]. It is important to note that the established indications for ICD placement include primary and secondary prevention of sudden cardiac death (SCD); primary prevention of SCD due to life-threatening ventricular tachycardia (VT) or ventricular fibrillation (VF) in patients who are optimally treated with medical management yet remain at an elevated risk of SCD and the secondary prevention of SCD in patients with a prior episode of resuscitated VT/VF or hemodynamically unstable VT in whom a completely reversible cause cannot be identified and in patients with episodes of spontaneous sustained VT in the presence of structural heart disease or channelopathy. Of note, ICD therapy is not indicated for the management of ventricular tachyarrhythmias due to electrolyte imbalance or drug use, which are deemed to be reversible causes [5].

Hence, multidisciplinary discussions were held to determine if it would be appropriate to implant an ICD in our patient. It was thought that the patient would benefit from ICD placement given her history of ongoing chronic diarrhea and malabsorption syndrome, which would be difficult to correct with peroral supplementation due to her bariatric surgery, underlying bradycardia preventing medical management with a beta-blocker, possible urinary electrolyte losses (from suspected Gitelman’s syndrome), and, most importantly, secondary prevention following her cardiac arrest on presentation.

This case also illustrates the risks of bariatric surgery in a patient with an underlying predisposition for long QT, where even a small shift in the electrolyte homeostasis can contribute to extremely deleterious consequences. To date, there is one reported case of a patient who underwent bariatric surgery and sustained an intraoperative cardiac arrest due to pulseless VT as a result of LQTS [6]. With the ever-increasing number of people undergoing bariatric surgery, it is important to risk stratify patients wherein even an insignificant-appearing electrolyte imbalance as a result of unavoidable consequences of bariatric surgery (such as malabsorption, dumping syndrome, diarrhea, gastroparesis ) could be detrimental, especially if a patient is on other QT-prolonging medications such as antidepressants, antipsychotics, and antihistamines. Of note, all patients undergoing bariatric surgery are at risk for acquired LQTS due to morbid obesity, 87% are female, and 50% have concomitant depression and are taking QTc prolonging antidepressants at the time of their surgery [7]. It is important to note that LQTS in morbidly obese patients is not a contraindication for bariatric surgery because of the benefits of bariatric surgery, including a reduction in LV mass and weight loss, both of which have shown to cause a reduction in the QT interval [8].

In summary, this case presented us with a unique diagnostic and therapeutic dilemma because the patient had an acquired long QT from an interplay of almost all the known predisposing reversible factors of QT prolongation, which ultimately lead to a VF cardiac arrest, thereby justifying ICD placement.

## Conclusions

LQTS, whether acquired or congenital, is characterized by disturbances in ventricular repolarization, leading to a prolonged QT interval, which puts patients at risk for life-threatening ventricular arrhythmias, including polymorphic ventricular tachycardia, typically torsades de pointes, which may deteriorate into ventricular fibrillation and lead to sudden cardiac death. This case report emphasizes the complexities involved in the management of LQTS, especially in the setting of multifactorial predisposing factors, including electrolyte abnormalities, malabsorption, QT-prolonging medication use, bariatric surgery, bradycardia, renal electrolyte losses, and so on. It also comments on the technical considerations involved in the placement of ICDs in such patients.
